# Decrease in Tripartite Motif Containing 24 suppresses hypoxia-induced proliferation and migration of pulmonary arterial smooth muscle cells via the AKT/mammalian target of rapamycin complex 1 pathway

**DOI:** 10.1080/21655979.2022.2080423

**Published:** 2022-06-02

**Authors:** Jingwen Xu, Yujia Zhong, Zhang Wang

**Affiliations:** Department of Geriatrics, The General Hospital of Western Theater Command, Chengdu, P.R. China

**Keywords:** TRIM24, pulmonary arterial hypertension, pulmonary artery smooth muscle cells, AKT, mTORC1

## Abstract

Tripartite Motif Containing 24 (TRIM24) is an oncogenic protein and promotes proliferation in several cancer cell lines. Nevertheless, the role of TRIM24 in proliferation and migration of pulmonary artery smooth muscle cells (PASMCs) remains to be clarified. The current study was aimed to reveal the role of TRIM24 in proliferation and migration of PASMCs during the development of pulmonary arterial hypertension (PAH). In pulmonary arteries (PAs) of chronic hypoxia-PAH (CH-PAH) mice and PASMCs challenged with hypoxia, the expression of TRIM24 was increased. Silencing TRIM24 by *Trim24* short hair RNA (shTrim24) suppressed hypoxia-induced increase in Ki-67 positive PASMCs and wound-healing rate. Furthermore, hypoxia caused enhanced phosphorylation of AKT and two major effectors of mammalian target of rapamycin complex 1 (mTORC1), S6 and 4EBP1 in PASMCs. The enhancement was then attenuated after silencing TRIM24. Both restoring mTORC1 activity and AKT reactivation abolished silencing TRIM24-mediated inhibition of proliferation and migration of PASMCs. Additionally, AKT reactivation also reversed the declined phosphorylation of S6 and 4EBP1 induced by shTrim24. In conclusion, TRIM24 is involved in the excessive proliferation and migration of PASMCs after hypoxic stimulus during PAH. The mechanism of TRIM24-mediated regulation of PASMCs is partly illustrated by promoting activity of AKT/mTORC1 signaling pathway.

## GRAPHICAL ABSTRACT

**Figure:**
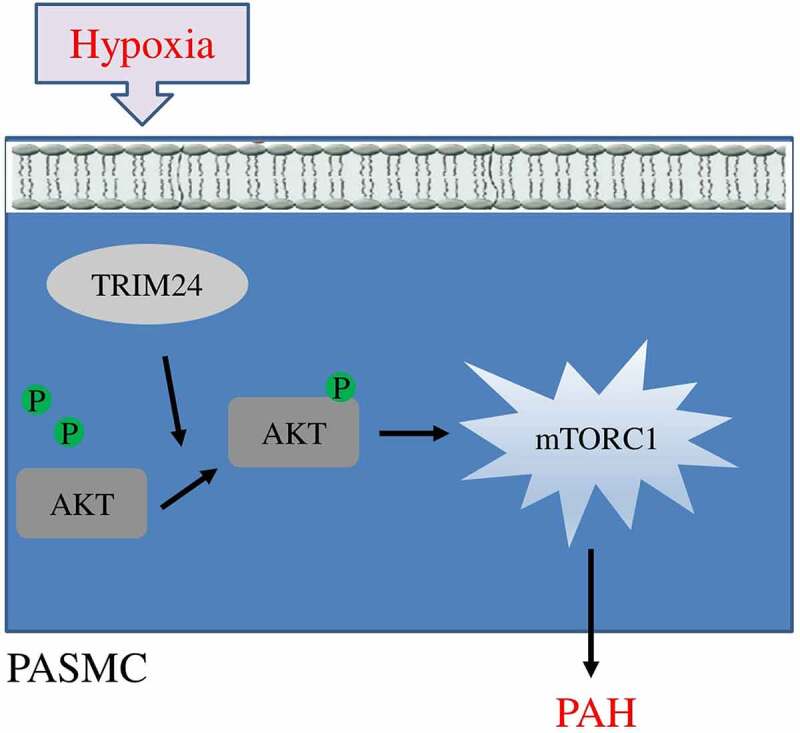

## Highlights

- TRIM24 is increased in hypoxia-treated PASMCs and PAs of CH-PAH mice.
- Silencing TRIM24 suppresses hypoxia-induced proliferation and migration of PASMCs.
- The activation of AKT/mTORC1 is responsible for TRIM24-mediated regulation on PASMCs.

## Introduction

Chronic pulmonary arterial hypertension (PAH) is a disease characterized by pathological pulmonary vascular remodeling and progressive pathological pulmonary artery (PA) contraction, resulting in high vascular resistance, right ventricular hypertrophy, and failure [1]. PAH is often complicated with chronic hypoxic diseases, such as interstitial lung disease, obstructive sleep apnea and chronic obstructive pulmonary disease [2]. Currently available drugs recovering the vasoconstrictive phenotype of PA mainly target vasoactive factors such as prostacyclin or endothelin-1. However, these drugs do not affect pulmonary vascular remodeling and thus fail to ultimately improve the outcomes of PAH patients [3]. Therefore, it is urgent to search new therapeutic strategies of PAH.

Pulmonary artery smooth muscle cells (PASMCs) are the main component of PA, thereby exerting a crucial role in physiological regulation of pulmonary vessel. Previous research demonstrates that chronic hypoxia-induced abnormal proliferation and migration of PASMCs is involved in the development of PAH and also associated with vascular stenosis and occlusion [4]. A variety of cellular and molecular mechanisms are involved in the modulation of PASMC proliferation and migration, including K^+^ channel, Ca^2+^ channel, transforming growth factor-β receptors and membrane receptors, etc [5]. However, the precise events that are involved in the enhanced proliferation and migration of PASMC remain unclear.

Tripartite Motif Containing 24 (TRIM24), also called TIF1α, belongs to the member of Transcription Intermediary Factor family. It consists of a terminal plant homeodomain (PHD)-bromodomain and a RING (E3 ubiquitin ligase) domain [6]. TRIM24 is abnormally expressed in multiple tumors and thus functions as oncogenic factors [7,8]. The tumor-promoting effect of TRIM24 is associated with its pro-proliferative role in different cancer cells. Additionally, TRIM24 is also confirmed to exert certain role in cardiovascular events. For example, TRIM24 can promote cardiomyocyte hypertrophy via regulation of dysbindin protein [9]. However, the role of TRIM24 in proliferation, migration of PASMC and PAH remains unknown.

AKT (also known as protein kinase B)/mammalian target of rapamycin (mTOR) is an important intracellular transduction system regulating cell proliferation, differentiation, migration, and protein synthesis [5]. mTOR exerts its role by forming two complexes, mTOR complex (mTORC)1 and mTORC2. AKT inhibits a crucial indirect regulator of mTORC1, the tuberous sclerosis complex 1 (TSC1) to activate mTORC1 [10]. Excessive AKT/mTORC1 activation is implicated in enhanced proliferation and migration of PASMCs [11]. TRIM24 can enhance the phosphorylation of AKT to induce proliferation of glioma cell proliferation [12]. It is unclear whether AKT/mTORC1 acts downstream of TRIM24 in PASMCs.

We hypothesized that TRIM24 might exert a regulatory role in the development of PAH. In the current study, we found TRIM24 was increased in PAs of chronic hypoxia-pulmonary arterial hypertension (CH-PAH) mice and hypoxia-challenged PASMCs. Silencing TRIM24 inhibited proliferation and migration of PASMCs via suppressing AKT/mTORC1 activity. This study aimed to search novel target for preventing PAH.

## Materials and methods

### Animals

C57BL/6 J mice obtained from Dashuo Animal Science and Technology (Chengdu, Sichuan, China). Mice were housed at room temperature in a 12-h light–dark cycle and have free access to water and food. CH-PAH mice model were established by using SU5416-hypoxia as previously described [13]. Briefly, male mice at the age of 8–10 weeks were subcutaneously injected with SU5416 (20 mg/kg; Sigma, St Louis, Missouri, USA) once a week and housed in a ventilated chamber with chronic hypoxia (10% O_2_) for 3 weeks. Normoxic control mice were subcutaneously injected with equivalent DMSO and kept in room air. All animal experiments in the study were performed in accordance with the Institutional Animal Care and Use Committee and the Ethic Committee of The General Hospital of Western Theater Command (2021EC1-7; Chengdu, Sichuan, China). Mice were finally anesthetized with pentobarbital (100 mg/kg) and sacrificed for other experiments.

### Detection of right ventricular systolic pressure (RVSP) and right ventricular hypertrophy index (RVHI)

RVSP and RVHI were measured as previously described [14]. Briefly, mice were anesthetized by intraperitoneal injection with pentobarbital (30 mg/kg). For determination of RVSP, a pressure transducer catheter (Millar Instruments, Houston, TX, USA) was inserted into the right ventricle. For RVHI, cardiac tissues of sacrificed mice were isolated and then the mass ratio of RV to left ventricle + septum was calculated as RVHI.

### Immunoblotting

Immunoblotting was performed as previously described [11]. The lysates of PAs and PASMCs were collected after treatment of RIPA buffer (Beyotime Institute of Biotechnology, Shanghai, China). Protein lysates were separated by gel electrophoresis and transferred to nitrocellulose membranes (Beyotime). The membranes were then blocked and subjected to primary antibodies against TRIM24 (cat ab256491, 1: 2000; Abcam; Cambridge, England), p-AKT^Thr308^ (cat 13038S, 1: 1000; Cell Signaling Technology; Danvers, Massachusetts, USA), AKT (cat 9272S, 1: 1000; Cell Signaling Technology), p-S6^Ser235/236^ (cat 4858S, 1: 1000; Cell Signaling Technology), S6 (cat 14467S, 1: 1000; Cell Signaling Technology), p-4EBP1^Thr37/46^ (cat 2855S, 1: 1000; Cell Signaling Technology), 4EBP1 (cat 9644 T, 1: 1000; Cell Signaling Technology), phosphoinositide 3-kinase (PI3K) p110α (cat 4255S, 1: 1000; Cell Signaling Technology), cyclin D1 (cat 2978S, 1: 1000; Cell Signaling Technology) and GAPDH (cat 4970S, 1: 8000; Cell Signaling Technology) overnight at 4°C. The membranes were next incubated with secondary antibodies.

### Cells

PASMCs were isolated and cultured as previously described [11]. Briefly, PAs were excised and kept in ice-cold PBS to remove their endothelium, adventitia, and connective tissues. The media were dissected, cut into small pieces and then incubated with Dulbecco’s modifed Eagle’s medium (DMEM; HyClone, Carlsbad, California, USA) filled with 10% fetal calf serum (Invitrogen, Carlsbad, California, USA), penicillin (100 units/mL) and streptomycin (100 mg/mL) at 37°C in a humidified 5% CO2 incubator. PASMCs between passages 3 and 5 was utilized for further experiments. Hypoxic PASMCs were incubated at 37°C under the condition of 3% O_2_, 92% N_2_ and 5% CO_2_ for 6, 12 and 24 hours (h), while normoxic PASMCs were cultured at 37°C under the condition of 21% O_2_, 74% N_2_ and 5% CO_2_ for the same time points. The adenovirus carrying *Trim24* short hairpin RNA (shTrim24) and control viruses (shCon) were constructed according to the manufacturer’s instructions (Genechem; Shanghai, China). For transfection in PASMCs, cells were transfected with relative adenovirus (3 pfu/cell) for 72 hours. For siRNA transfection, PASMCs were incubated with the mixture of control siRNA or *Tsc1* siRNA (siTsc1) and 10 μL X-tremeGENE siRNA transfection reagent for 8 h. Then, PASMCs were cultured for another 48 h before experiments. The sequence of siTsc1: GCUUUGACUCUCCCUUCUA [15]. SC-79 (10 μM; MCE, Shanghai, China) was used to treat PASMCs for 24 h [16].

### Immunofluorescence staining

Immunofluorescence staining of PASMC was performed as previously described [11]. PASMCs were culturex in a 6-well plate, fixed with 4% polyformaldehyde and then incubated with 0.5% triton X-100 T to break the membrane. After being blocked by a blocking solution, PASMCs were next incubated in dark with a primary antibody against Ki-67 (1:1000; Cell Signaling Technology) at 4°C overnight. PASMCs were then washed by PBS and incubated with a secondary antibody in dark for 1 h. Finally, the nuclei were stained by using DAPI (5 mg/ml; VECTOR Labs, Burlingame, California, USA) at 25°C for 5 s. Images were obtained with an immunofluorescent microscopy (Leica MPS 60, Germany).

### Wound-healing assay

Wound-healing assay was performed as a previous study described [15]. PASMCs were cultured on a 6-well plate and grown to 90% confluency. PASMCs were serum-deprived for 12 h and scratches were made by using a 10-μL sterile pipette in the middle area of cells. PASMCs were then cultured under normoxic or hypoxic condition for 24 h. The images were taken in real time from 0 to 24 hours. The rates of wound closure were directly measured by using microscopic visualization and followed with a reference point at the bottom of the plate to capture the same spot every time.

### Statistical analysis

Comparisons between two groups were conducted by Student’s *t*-test. Comparisons between multiple groups were conducted by analysis of variance (ANOVA) with an appropriate *post hoc* test. P < 0.05 was considered to be statistically significant. All data are expressed as mean ± standard deviation (SD.).

## Results

In our current study, we explored the role of TRIM24 in proliferation and migration of PASMCs by utilizing the adenovirus carrying shTrim24. We demonstrated that silencing Trim24 suppressed hypoxia-induced proliferation and migration in PASMCs in an AKT/mTORC1-dependent manner.

### The protein expression levels of TRIM24 in CH-PAH mice and hypoxic PASMCs were increased

**Figure 1.**
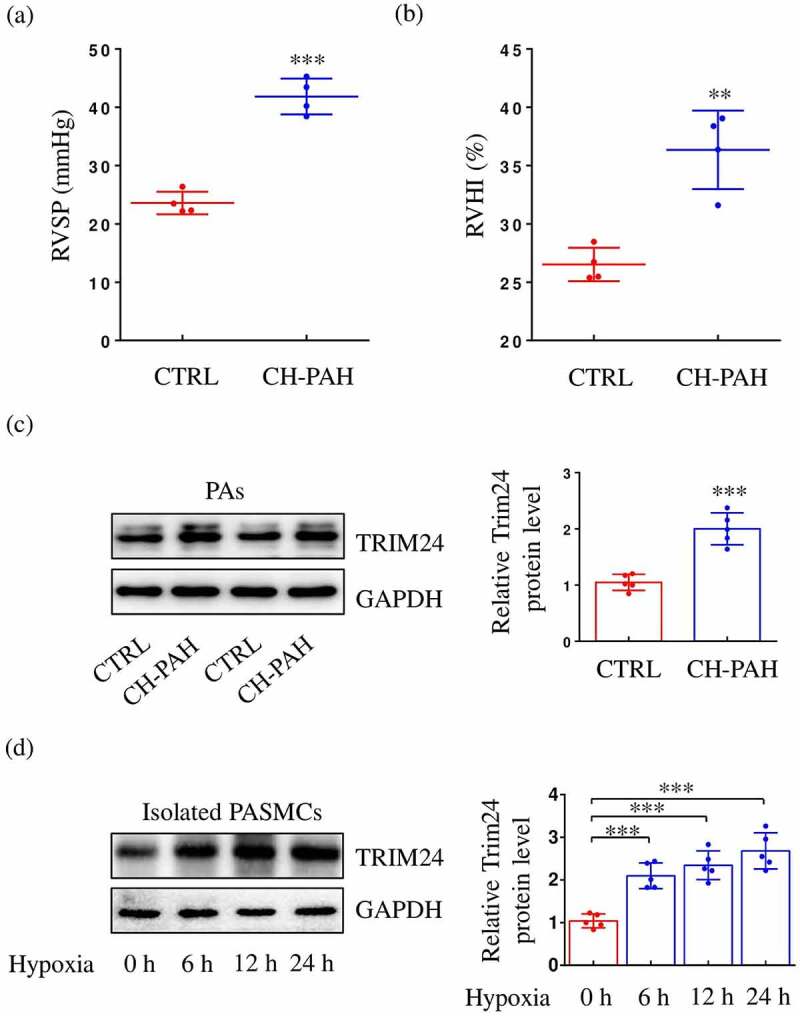
The expression of TRIM24 is increased in pulmonary arteries (PAs) of chronic hypoxia-pulmonary arterial hypertension (CH-PAH) mice and hypoxia-treated PASMCs. RVSP (a) and RVHI (b) of CH-PAH mice were analyzed (n = 4). RVSP: right ventricular systolic pressure. RVHI: right ventricular hypertrophy index. (c) The relative protein level of TRIM24 in dissected PAs of CH-PAH mice was measured by immunoblotting (n = 5). (d) The relative protein level of TRIM24 in PASMCs exposed to hypoxic conditions for different time courses was measured by immunoblotting (n = 5). Data are expressed as mean ± SD. ** and *** indicate a significant difference of P < 0.01 and P < 0.001 between the two marked groups, respectively.

To investigate whether TRIM24 is implicated in the modulation of PAHs, we first determined the expression of TRIM24 in PAs of CH-PAH mice. The model of CH-PAH mice was confirmed by increased RVSP (Figure 1(a)) and RVHI (Figure 1(b)). We found that PAs in CH-PAH mice displayed significantly higher TRIM24 expression than PAs in control mice (Figure 1(c)). Hypoxia induces excessive proliferation and migration of PASMCs, thus accelerating the development of PAH [4]. We thus determined the expression of TRIM24 in hypoxia-challenged PASMCs. Our data showed that 6, 12, and 24 hours of hypoxia exposure both led to significantly increased expression of TRIM24 (Figure 1(d)). Therefore, upregulation of TRIM24 might play a crucial role in the regulation of abnormal proliferation, migration of PASMCs, and PAH.

### Silencing TRIM24 inhibits hypoxia-induced proliferation and migration of PASMCs

**Figure 2.**
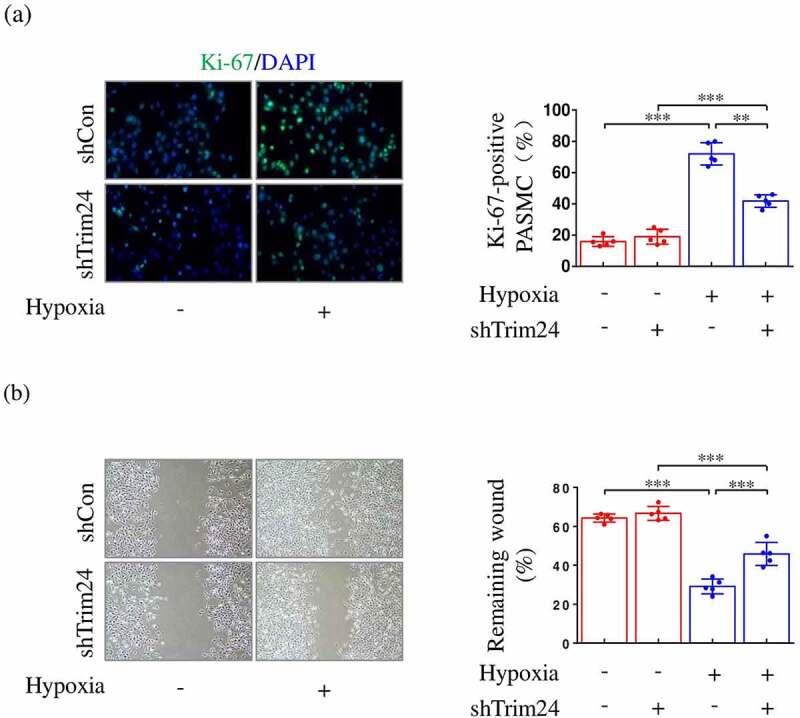
Silencing TRIM24 inhibits hypoxia-induced proliferation and migration of PASMCs. PASMCs were transfected with the adenovirus carrying *Trim24* shRNA (shTrim24) or control virus (shCon) and incubated under normoxic/hypoxic conditions for 24 h. (a) Ki67 (green) and DAPI (blue) staining was performed by immunofluorescence staining (n = 5). Magnification 400 × . (b) Cell migration was analyzed by wound healing assay (n = 5). Magnification 100 × . Data are expressed as mean ± SD. ** and *** indicate a significant difference of P < 0.01 and P < 0.001 between the two marked groups, respectively.

To investigate the relationship of TRIM24 upregulation with increased proliferation and migration of PASMCs after hypoxic stimulus, we next used shTrim24 to silence the expression of TRIM24 and analyzed the changes of proliferation and migration of PASMCs. By using Ki-67 immunofluorescent staining, we found that 24 h-treatment with hypoxia significantly increased the number of Ki-67 (a marker of cell proliferation) positive PASMCs compared to normoxic treatment. However, the elevation of Ki-67 positive cell number was obviously reversed after silencing TRIM24 (Figure 2(a)). Subsequent experiments were performed to investigate whether the capacity of migration was also influenced by TRIM24 in PASMCs. In the current study, PASMC migration was analyzed by *in vitro* scratch assay. Hypoxia treatment accelerated the rate of wound-healing compared to normoxic condition. Simultaneously, the increased wound-healing rate of PASMCs was slowed down once TRIM24 was silenced (Figure 2(b)). These data indicate that downregulation of TRIM24 can effectively suppress hyperproliferation and hypermigration in PASMCs after hypoxic stimulus.

### Silencing TRIM24 inhibits proliferation and migration of PASMCs via reducing mTORC1 activity

We next tried to reveal the molecular mechanism by which TRIM24 promoted proliferation and migration of PASMCs. It was shown that both excessive proliferation and migration of PASMCs and the development of PAH were associated with a significant increase in mTORC1 activity [11]. Whether TRIM24 regulates the activity of mTORC1 in PASMCs is unknown.

**Figure 3.**
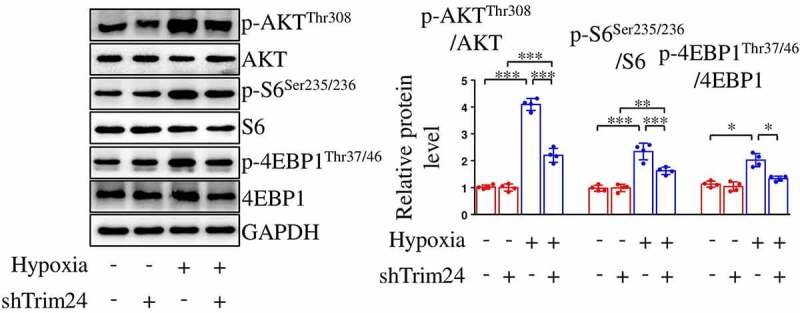
Silencing TRIM24 suppresses hypoxia-induced phosphorylation of AKT, S6 and 4EBP1 in PASMCs. PASMCs were transfected with shTrim24 or shCon and incubated under normoxic/hypoxic conditions for 24 h. The relative protein levels of p-AKT^Thr308^, AKT, p-S6^Ser235/236^, S6, p-4EBP1^Thr37/46^ and 4EBP1 in PASMCs were analyzed by using immunoblotting (n = 4). Data are expressed as mean ± SD. *, ** and *** indicate a significant difference of P < 0.05, P < 0.01 and P < 0.001 between the two marked groups, respectively.

**Figure 4.**
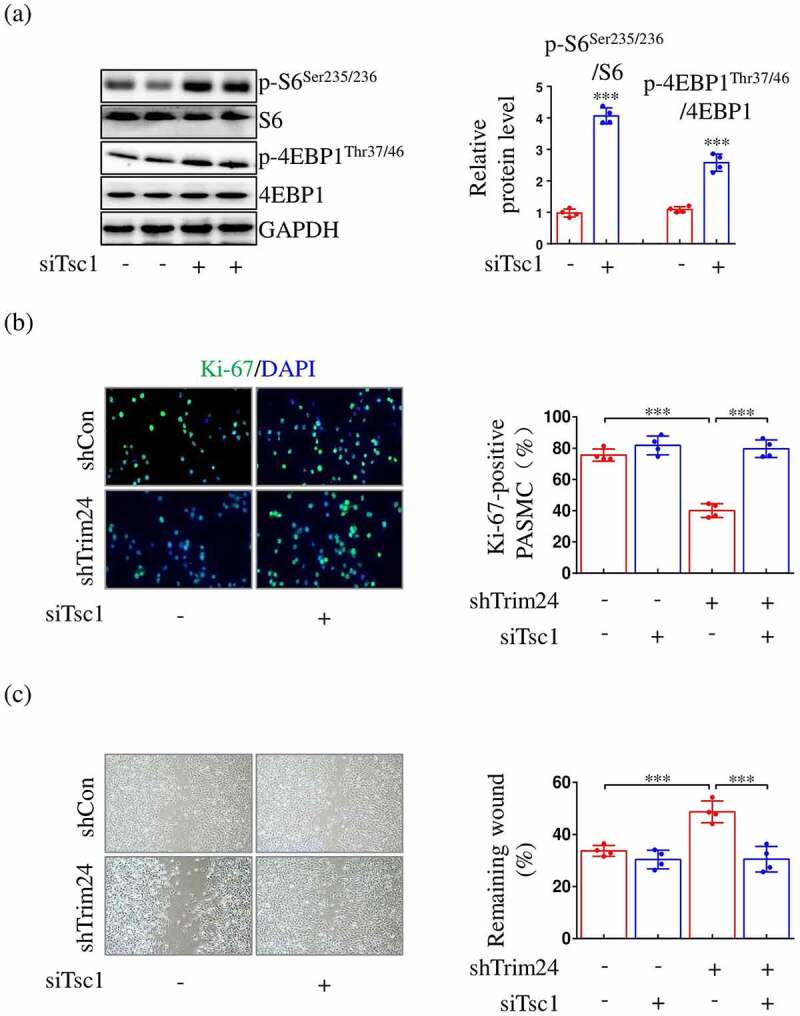
Restoring mTORC1 activity abolishes shTrim24-induced decrease in proliferation and migration of PASMCs exposed to hypoxia. PASMCs were firstly treated with incubated with siCon or siTsc1 for 8 h. Then, cells were transfected with shTrim24 or shCon and cultured under normoxic/hypoxic conditions for 24 h. (a) The protein expression levels of p-S6^Ser235/236^, S6, p-4EBP1^Thr37/46^ and 4EBP1 in PASMCs transfected with siCon or siTsc1 were evaluated by immunoblotting. Representative bands (left panel) and corresponding quantification (right panel) were shown (n = 4). (b) Ki67 (green) and DAPI (blue) staining was performed by immunofluorescence staining (n = 4). Magnification 400 × . (c) Cell migration was analyzed by wound healing assay (n = 4). Magnification 100 × . Data are expressed as mean ± SD. *** indicates a significant difference of P < 0.001 between the two marked groups.

S6 and 4EBP1 are two major downstream effectors of mTORC1. Therefore, the phosphorylation levels of the two proteins reflect the activity of mTORC1. As shown in our data, the phosphorylation levels of S6 and 4EBP1 in PASMCs were both increased after hypoxic stimulus (Figure 3). Moreover, the accelerated phosphorylation of S6 and 4EBP1 induced by hypoxia was partly rescued after shTrim24 transfection (Figure 3). To investigate whether increased mTORC1 activity was associated with excessive proliferation and migration of PASMCs, we utilized siRNA of a classical gene inhibiting mTORC1 activity, tuberous sclerosis complex (siTsc1) to recover the reduced mTORC1 activity in hypoxia-challenged PASMCs (Figure 4(a)). The results demonstrated that the decrease in the proliferation and migration of TRIM24-silenced PASMCs reversed when the cells were transfected with siTsc1 (Figure 4(b,c)).

### Silencing TRIM24 suppresses mTORC1 activity in PASMCs by inhibiting AKT phosphorylation

We next identified the signaling pathways that underlie mTORC1 inactivation in PASMCs after shTrim24 transfection. Serving as a sensor for cellular stress and energy metabolism, mTORC1 is affected by varies of stress-associated kinases such as PI3K/AKT [17]. TRIM24 is proved to be an activator for AKT activation in several cancer cell lines [12,18]. Whether TRIM24 activates AKT to increase mTORC1 activity in PASMCs is unrevealed.

**Figure 5.**
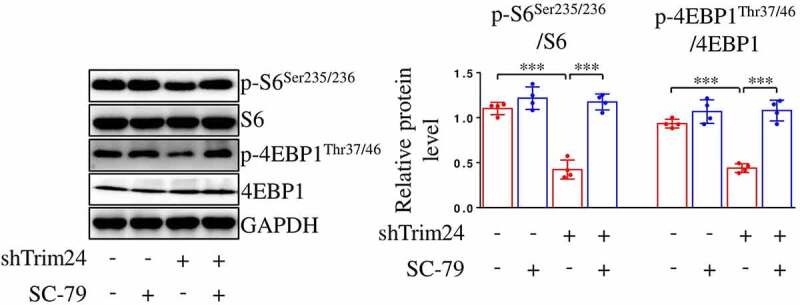
TRIM24-mediated regulation of mTORC1 in PASMCs dependents on AKT. PASMCs were firstly incubated with SC-79 (AKT agonist; 10 μM) or equivalent vehicle for 24 h. Then, cells were transfected with shTrim24 or shCon and cultured under hypoxic condition for another 24 h. Images of immunoblotting and relative expression levels of p-S6^Ser235/236^, S6, p-4EBP1^Thr37/46^ and 4EBP1 in PASMCs were shown (n = 4). Data are expressed as mean ± SD. *** indicates a significant difference of P < 0.001 between the two marked groups.

As illustrated in Figure 3, hypoxia enhanced the phosphorylation of AKT at site Thr308 in PASMCs, while silence of TRIM24 abolished the increase in AKT phosphorylation (Figure 3). Next, we utilized an agonist of AKT, SC-79 to rescue the declined activity of AKT induced by shTrim24. We found that silencing TRIM24 failed to attenuate the phosphorylation of S6 and 4EBP1 in PASMCs after application of SC-79 (Figure 5). However, silencing TRIM24 did not affect the expression of PI3K p110α in PASMCs (Supplementary Figure S1), indicating PI3K was not involved in TRIM24-mediated regulation of mTORC1. These data identify that TRIM24 silence-induced mTORC1 inactivation in PASMCs is mediated by AKT dephosphorylation.

### AKT/mTORC1 act downstream of TRIM24 to induce proliferation and migration of PASMCs

**Figure 6.**
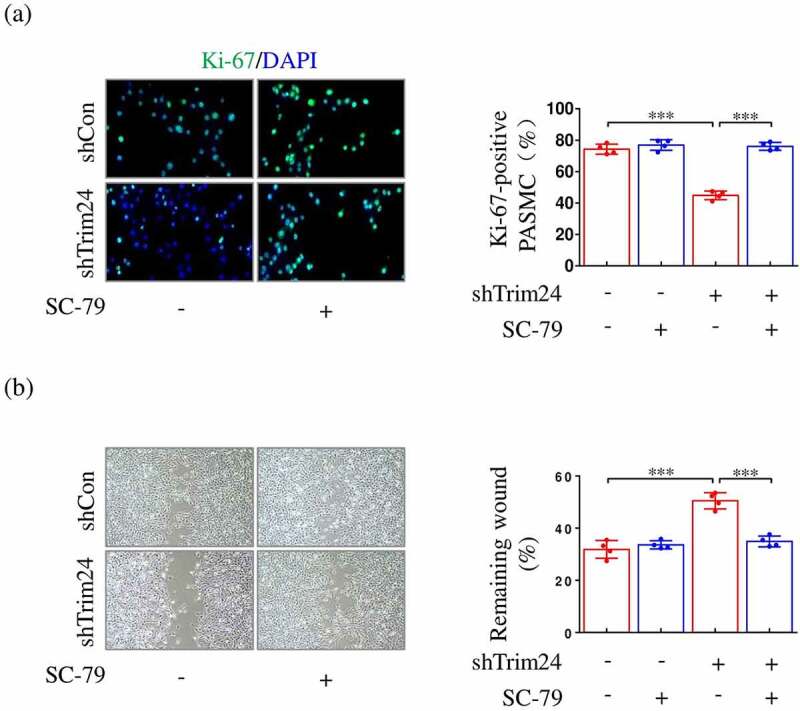
AKT activation reverses shTrim24-induced decrease in proliferation and migration of hypoxia-challenged PASMCs. PASMCs were firstly incubated with SC-79 (AKT agonist; 10 μM) or equivalent vehicle for 24 h. Then, cells were transfected with shTrim24 or shCon and cultured under hypoxic condition for another 24 h. (a) Ki67 (green) and DAPI (blue) staining was performed by immunofluorescence staining (n = 4). Magnification 400 × . (b) Cell migration was analyzed by wound healing assay (n = 4). Magnification 100 × . Data are expressed as mean ± SD. *** indicates a significant difference of P < 0.001 between the two marked groups.

Published observations confirm the important role of AKT/mTORC1 axis in proliferation and migration of PASMCs [5]. Since restoring AKT activity abolished shTrim24-mediated suppression of mTORC1 activity, we investigated whether AKT inhibition also impaired shTrim24-mediated regulation on proliferation and migration of PASMCs. The results showed that the proportion of Ki-67 positive PASMCs was significantly reduced in hypoxic-challenged PASMCs that was transfected with shTrim24 compared to shCon. Simultaneously, AKT reactivation by SC-79 significantly attenuated the decrease in the number of Ki-67 positive PASMCs induced by silencing TRIM24 (Figure 6(a)). We also determined the expression of cyclin D1, a cell cycle regulator downstream of AKT in PASMCs [19]. We found that silencing TRIM24 inhibited the expression of cyclin D1 in hypoxia-challenged PASMCs, which was reversed by SC-79 (Supplementary Figure S2). Decreased migration of TRIM24-silenced PASMCs was also demonstrated by wound healing assay. However, the attenuated capacity of cell migration induced by TRIM24 inhibition was also reversed after SC-79 utilization (Figure 6(b)). Taken together, these findings suggest that AKT dephosphorylation-mediated mTORC1 inactivation by silencing TRIM24 suppresses excessive proliferation and migration of PASMCs.

## Discussion

The current study was aimed to reveal the regulatory role of TRIM24 in the proliferation and migration of PASMCs and the underlying mechanism. The protein level of TRIM24 was elevated in both PAs of CH-PAH mice and PASMCs after hypoxic treatment. Silencing TRIM24 *in vitro* reduced proliferation and migration of PASMCs challenged with hypoxia. In PASMCs, TRIM24 inhibition reduced AKT phosphorylation and mTORC1 activity. AKT reactivation and restoring mTORC1 activity both reversed silencing TRIM24-mediated regulation of PASMCs, which provided novel insight into the regulatory role and mechanism of TRIM24 in proliferation and migration of PASMCs and revealed a potential therapeutic target for PAH.

TRIM24 is a member of the transcription intermediary factor family and plays important roles in T cell differentiation, innate immune regulation, and tumor development [20]. TRIM24 is also reported to be also implicated in cardiovascular regulation, such as cardiomyocyte hypertrophy [9]. We found the expression of TRIM24 was significantly increased in PAs of CH-PAH mice, reminding us that TRIM24 might play a certain role in the regulation of PAH. Hypoxia-associated abnormal proliferation and migration of PASMCs is an crucial step of pathological vascular remodeling during PAH [1]. Therefore, we also confirmed the expression of TRIM24 in hypoxia-treated PASMCs and observed increased TRIM24 after hypoxic stimulus. In accordance with other cancer cell lines [7,8], silencing TRIM24 by shTrim24 also attenuated hypoxia-induced proliferation and migration of PASMCs. These results showed that TRIM24 might partly illustrate why proliferation and migration of PASMCs was enhanced during the development of PAH. Nevertheless, the exact downstream biological mechanism of TRIM24 remains unclear.

mTORC1 is a crucial serine/threonine kinase and implements regulatory roles in cell growth, proliferation, and differentiation, etc [5]. mTORC1 has also been established as a major mechanism for the development of PAH [5]. For example, mTORC1 over-activation is associated with endothelial dysfunction [21], pathological PASMC proliferation [11], and PASMC-endothelial cell communication [22], thus accelerating the progression of PAH. TRIM24 leads to mTORC1 activation in mouse mammary epithelia [23]. Consistently, we found that silencing TRIM24 also significantly inhibited mTORC1 activity in hypoxic-challenged PASMCs. The inhibitory roles of shTrim24 in proliferation and migration of PASMCs were abolished after restoring mTORC1 activity. Therefore, mTORC1 is essential for the suppressive effects of silencing TRIM24 on proliferation and migration of PASMCs.

The PI3K/AKT is a classical signaling pathway that regulates proliferation, migration of PASMCs and PAH [24]. mTORC1 is a major downstream effector of PI3K/AKT pathway. AKT positively regulates mTORC1 activity via affecting its many kinases, such as suppressing TSC1/2 [10] or promoting phosphorylation of PRAS40 [25]. Our current study showed that AKT phosphorylation at site Thr308 in PASMCs was also obviously decreased by silencing TRIM24. Additionally, shTrim24-induced declined phosphorylation of S6 and 4EBP1 was also rescued after AKT reactivation. Therefore, we proposed that AKT might also act downstream of TRIM24 to activate mTORC1 in PASMCs. Consistent with our hypothesis, the attenuated proliferation and migration of PASMCs induced by silencing TRIM24 was also reversed after reactivation of AKT. We thus confirmed that TRIM24 promoted hypoxia-induced proliferation and migration of PASMCs via enhancing AKT/mTORC1 activity.

The current study also has some limitations. For example, how TRIM24 affects AKT phosphorylation is still unclear. In addition, though we have confirmed the role of TRIM24/AKT/mTORC1 axis in proliferation and migration of PASMCs, whether this signaling pathway works in humans remains unknown. These doubts need further investigation in our future study.

## Conclusion

In summary, our findings identify TRIM24 as a potential target for the prevention and treatment of PAH. We first demonstrated that TRIM24 was increased in hypoxia-challenged PASMCs and PAs of CH-PAH mice. Decrease in TRIM24 abolished hypoxia-associated proliferation and migration of PASMCs, indicating hypoxia-induced upregulation of TRIM24 was responsible for excessive proliferation and migration of PASMCs. Next, we found mTORC1 signaling was activated by TRIM24 and acted downstream of TRIM24 to promote proliferation and migration of PASMCs. Our data also revealed a mechanism by which TRIM24 activated mTORC1 in PASMCs through phosphorylating AKT. The newly elucidated roles of TRIM24 in PASMCs also help us better understand the pathological mechanisms of PAH development.

## Supplementary Material

Supplemental Material
