# Supplementary material for: Decrease in Tripartite Motif Containing 24 suppresses hypoxia-induced proliferation and migration of pulmonary arterial smooth muscle cells via the AKT/mammalian target of rapamycin complex 1 pathway
Source: Bioengineered. 2022 Jun 2;13(5):13596–606. doi: 10.1080/21655979.2022.2080423 (PMC9275953; doi:10.1080/21655979.2022.2080423)
Supplement: Supplemental Material [file KBIE_A_2080423_SM7649.zip › 20220509_trim24_Supplementary information.docx]

**Decrease in Tripartite Motif Containing 24 suppresses hypoxia-induced proliferation and migration of pulmonary arterial smooth muscle cells via the AKT/mammalian target of rapamycin complex 1 pathway**

Jingwen Xu^a,*^, Yujia Zhong^a,*^, Zhang Wang^a^

**Supplementary information**

**Supplementary Figure Legends**

Supplementary Figure S1. Silencing TRIM24 does not affect PI3K expression in PASMCs. PASMCs were transfected with shTrim24 or shCon and incubated under normoxic/hypoxic conditions for 24 h. The relative protein level of PI3K p110α in PASMCs was analyzed by using immunoblotting (n = 4). Data are expressed as mean ± SD. *** indicates a significant difference of P < 0.001 between the two marked groups.

Supplementary Figure S2. AKT phosphorylation abolishes shTrim24-mediated inhibition of cyclin D1 expression in PASMCs. PASMCs were firstly incubated with SC-79 (AKT agonist; 10 μM) or equivalent vehicle for 24 h. Then, cells were transfected with shTrim24 or shCon and cultured under hypoxic condition for another 24 h. Images of immunoblotting and relative expression level of cyclin D1 in PASMCs were shown (n = 4). Data are expressed as mean ± SD. *** indicates a significant difference of P < 0.001 between the two marked groups.
