# Supplementary figures and images for: Decrease in Tripartite Motif Containing 24 suppresses hypoxia-induced proliferation and migration of pulmonary arterial smooth muscle cells via the AKT/mammalian target of rapamycin complex 1 pathway
Source: Bioengineered. 2022 Jun 2;13(5):13596–606. doi: 10.1080/21655979.2022.2080423 (PMC9275953; doi:10.1080/21655979.2022.2080423)

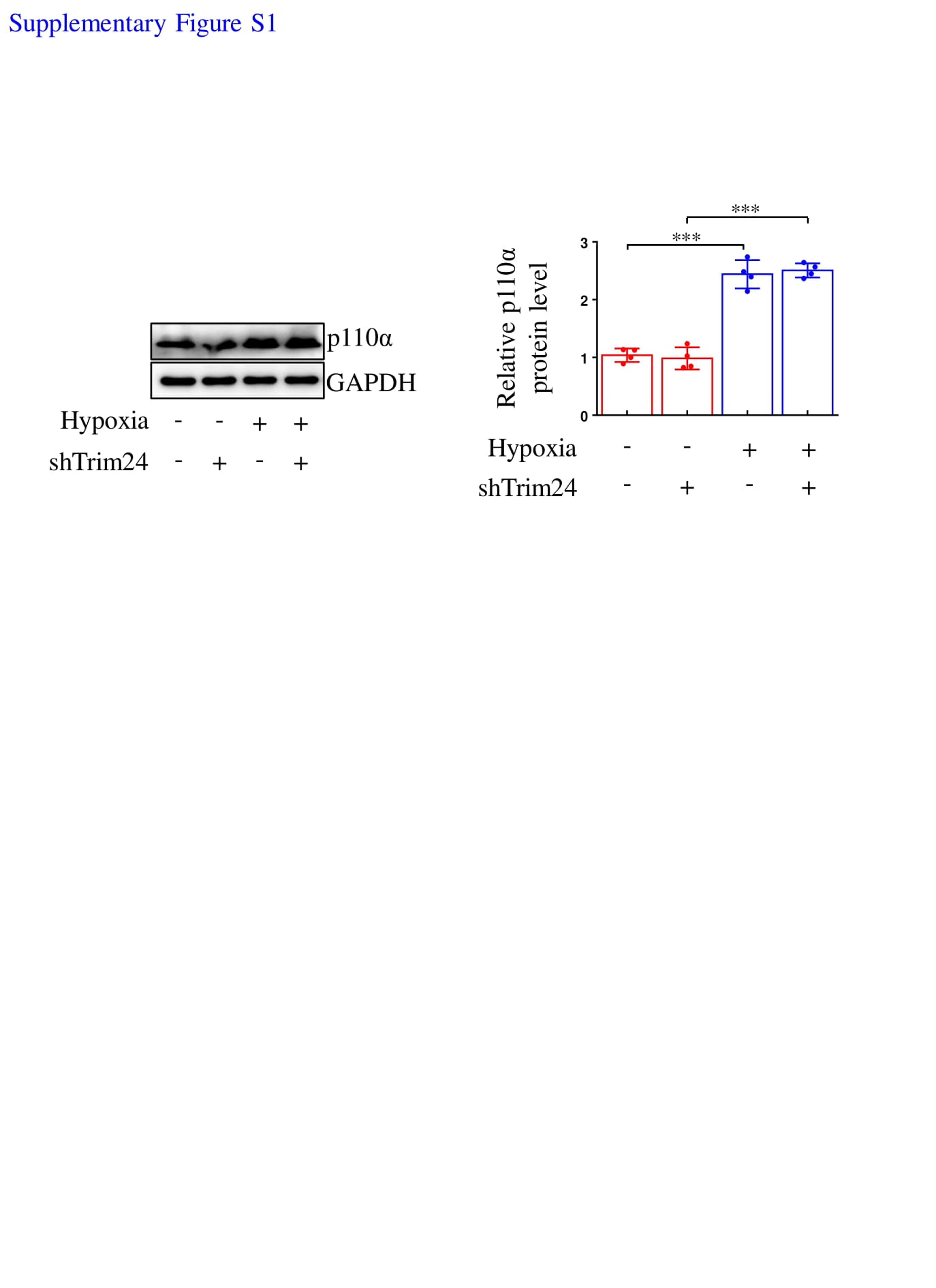

Supplement: Supplemental Material [file KBIE_A_2080423_SM7649.zip › Supplementary Figure S1.jpg]

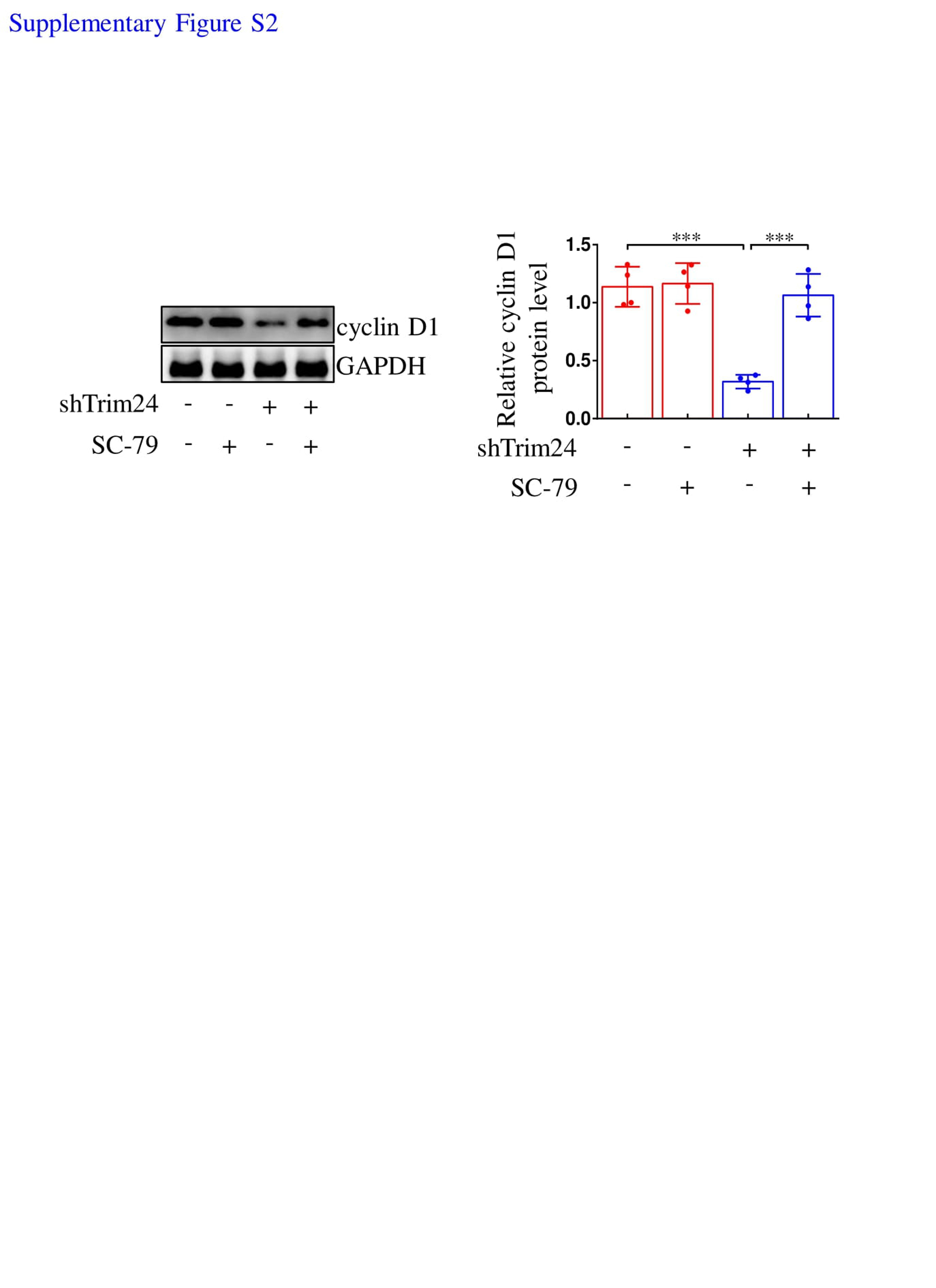

Supplement: Supplemental Material [file KBIE_A_2080423_SM7649.zip › Supplementary Figure S2.jpg]
